# The distance between new and previous incisions does not affect skin necrosis in total knee arthroplasty: a parallel-randomized controlled clinical trial

**DOI:** 10.1186/s12893-022-01791-w

**Published:** 2022-09-26

**Authors:** Ali Yeganeh, Mehdi Moghtadaei, Alireza Ghaznavi, Nader Tavakoli, Mohammad Soleimani, Sahand Cheraghiloohesara, Nima Taheri

**Affiliations:** 1 Department of Orthopedic Surgery, Rasoul-e-Akram Hospital, Iran University of Medical Sciences, Tehran, Iran; 2 Trauma and Injury Research Center, Hazrat-e Rasool Hospital, Iran University of Medical Sciences, Niayesh St, Satarkhan Av, Tehran, Iran; 3 Department of Epidemiology, School of Public Health, Iran University of Medical Sciences, Tehran, Iran

**Keywords:** Total knee arthroplasty, Surgical incision, Scar, Skin necrosis, Wound healing

## Abstract

**Background:**

To avoid skin necrosis, an 8 cm distance between the new and previous incision is recommended in patients undergoing total knee arthroplasty (TKA). It was hypothesized that making a new incision less than 8 cm of the prior scar does not increase the risk of skin complications, and the new incision can be made anywhere, regardless of the distance from the previous scar. This study investigated how making a new incision, irrespective of the previous scars, affects skin necrosis.

**Methods:**

In this parallel, randomized clinical trial, by simple randomization method using a random number table, 50 patients with single longitudinal knee scars were randomly assigned to two groups with a 1:1 ratio and 25 participants in each group. Patients with a minimum age of 60 and a single longitudinal previous scar on the knee were included. The exclusion criteria were diabetes mellitus, hypertension, morbid obesity, smoking, vascular disorders, cardiopulmonary disorders, immune deficiencies, dementia, and taking steroids and angiogenesis inhibitors. TKA was performed through an anterior midline incision, regardless of the location of the previous scar in the intervention group. TKA was performed with a new incision at least 8 cm distant from the old incision in the control group. Skin necrosis and scar-related complications were evaluated on the first and second days and first, second, and fourth weeks after the surgery. Knee function was assessed using the Knee Society Score (KSS) six months after the surgery.

**Results:**

The baseline characteristics of the groups did not differ significantly. The average distance from the previous scar was 4.1 ± 3.2 cm in the intervention group and 10.2 ± 2.1 cm in the control group. Only one patient in the control group developed skin necrosis (P-value = 0.31). Other wound-related complications were not observed in both groups. The mean KSS was 83.2 ± 10.2 and 82.9 ± 11.1 in the intervention and control groups, respectively (P-value = 0.33).

**Conclusions:**

It is possible that in TKA patients, the new incision near a previous scar does not increase the risk of skin necrosis and other complications.

## Background

Wound complications can occur in up to 20% of the cases after total knee arthroplasty (TKA) [1, 2]. Also, poor healing of the incisions can lead to potentially devastating results such as limb amputation [3]. Previous incision scars around the knee may complicate subsequent TKA procedures due to their susceptibility to skin complications such as poor wound healing, sepsis, and necrosis [4]. Hence, a minimum of 8 cm distance is recommended between new incisions and the previous scar [5, 6].

However, this distance can cause difficulties accessing the target structures in the knee. Also, a vertical incision is suggested in patients with multiple old scars, even if it leads to lateral arthrotomies [7–9]. It is also recommended to reuse the old longitudinal incisions [10]. Such suggestions all make the procedure more difficult for the surgeon.

In the two-stage fasciocutaneous flap, after the skin incision, it can be used as a flap, even three weeks after the removal, and any displacement does not result in skin necrosis [11]. Accordingly, it was hypothesized that making a new incision less than 8 cm from the previous scar does not increase the risk of skin complications, and the new incision can be made at the desired location, regardless of the distance of the prior scar. In this trial, the skin complications rate among the TKA patients with a single previous scar on whom a new incision was made based on the 8 cm-standard approaches or closer distance were compared.

## Methods

The ethics committee approved this randomized controlled clinical trial study of the Iran University of Medical Sciences with approval ID of IR.IUMS.REC.1399.1450. The first patient was registered on 08/08/2021. The study protocol was registered on the Iranian Registry of Clinical Trials with the number code: (IRCT20180528039883N2). All patients were informed of the study protocol and provided written consent informed prior to participation in the study. The recruitment period was from 21 June 2021 to February 2022 in Tehran, Iran. All patients selected for TKA were evaluated during the recruitment period for this study.

**Fig. 1:**
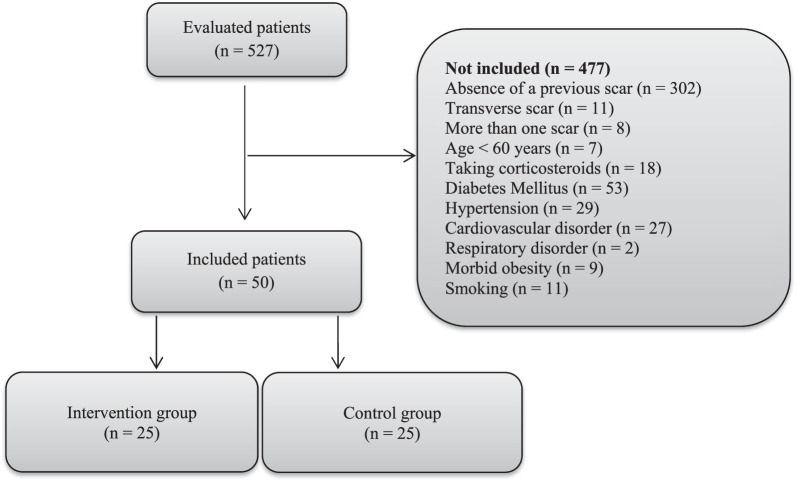
Flow diagram of the study inclusion and exclusion

The inclusion criteria were a minimum age of 60 years and a single longitudinal previous scar on the knee. Exclusion criteria were conditions affecting wound healing, including diabetes mellitus, hypertension, morbid obesity (BMI ≥ 40 kg/m^2^), smoking, vascular disorders, cardiopulmonary disorders, immune deficiencies, and dementia [12]. Also, patients taking medications with adverse effects on wound healing, such as steroids and angiogenesis inhibitors, were excluded. A flow diagram demonstrates the inclusion and exclusion of the patients (Fig. 1).

### Sample size

By considering a 95% confidence level, 80% power, and 20% dropout rate, a sample size of 25 in each group was calculated [13].

#### Randomization

The randomization was through a computer-generated random number list (Rand function of Excel software). Patients were randomly assigned to two groups in a ratio of 1:1. Randomization was performed by simple randomization method using a random number table.

#### Intervention

**Fig. 2:**
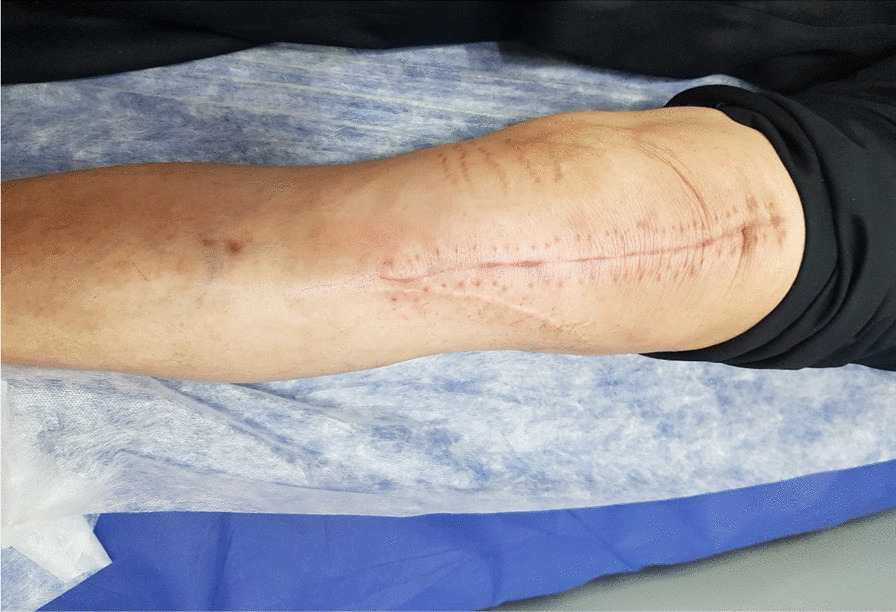
A distance of less than 8 cm between the new and previous incisions

The perioperative conditions of the two groups were kept similar. All patients were placed in the supine position, and under general anesthesia and inflation of a pneumatic tourniquet, a standard cemented TKA was implemented for all patients [14]. The same senior knee surgeon performed all surgeries, and posterior-stabilized prostheses (Zimmer, Warsaw, IN, USA) were used in all procedures. In the intervention group, an anterior midline incision was made regardless of the location of the previous scar (Fig. 2). The new incision was made at least 8 cm distant from the previous scar in the control group. After the operation, 10 mg oral once-daily rivaroxaban for up to 14 days was prescribed as a prophylaxis for venous thromboembolism. Also, the same postoperative analgesics were administered to all patients. The type, dose, and duration of antibiotic use for postoperative prophylaxis were similar in the two groups to control the confounder. The dressing was changed for the first 48 h, then changed every 48 h. The patients were routinely visited following the surgery.

#### Outcome measures

Primary outcome measures were the incidence of skin necrosis and other wound healing complications, e.g., wound hematoma, infection, and dehiscence. Wound complications such as necrosis, bleeding, infection, and abnormality in healing were investigated through the routine monitoring of the incision site during the follow-up visits, which were planned to be performed on the first and second days and the first, second, and fourth weeks after surgery. Skin necrosis was assessed by clinical judgment and regarded as a dichotomous condition. The secondary outcome measure was the knee function assessed with the Knee Society Score (KSS). A maximum score of 100 showed the best possible function, which was evaluated six months after the operation.

#### Statistical analysis

Statistical analysis was performed using SPSS for Windows, version 16 (SPSS Inc., Chicago, Ill., USA). A Kolmogorov–Smirnov test evaluated the normal distribution of data. An independent t-test was used to compare mean values between the groups. In non-parametric instances, the Mann–Whitney U test was used instead. A Pearson's chi-squared test was utilized to compare the qualitative variables. The P-value < 0.05 was considered statistical significant.

## Results

All 25 patients in each group received their allocated treatment, and no patient was lost. The average distance from the previous scar was 4.1 ± 3.2 cm in the intervention group, ranging from 2 to 7 cm (Fig. 2). This distance was 10.2 ± 2.1 cm in the control group, ranging from 8 to 13 cm. The mean preoperative KSS of the patients was not significantly different in the two study groups (P-value = 0.29). Also, There was no significant difference in the features of the previous scars in the two groups.(p > 0.05) (Table 1).

**Table 1:** Baseline characteristics and features of the previous scars in the two groups

Variable	< 8 cm distance between incisions (n = 25)	> 8 cm distance between incisions (n = 25)	P-value
Age (year)	65.5 ± 5.2)	64.8 ± 4.6	0.33
Sex			0.66
Male	3(12%)	5(20%)	
Female	22(88%)	20(80%)	
Laterality			0.83
Right	15(60%)	14(56%)	
Left	10(40%)	11(44%)	
Body mass index (kg/m^2^)	26.9 (2.4)	27.2 (3.0)	0.81
Type of previous surgery			0.52
Osteotomy	18(72%)	16(64%)	
Fracture	7(28%)	9(36%)	
Time interval from the previous surgery (years)	2.1 ± 1.1	2.4 ± 1.06	0.39
Previous incision length (cm)	7.2 ± 2.5	6.9 ± 2.7	0.41

P-value < 0.05 is considered significant

### Complications

**Fig. 3:**
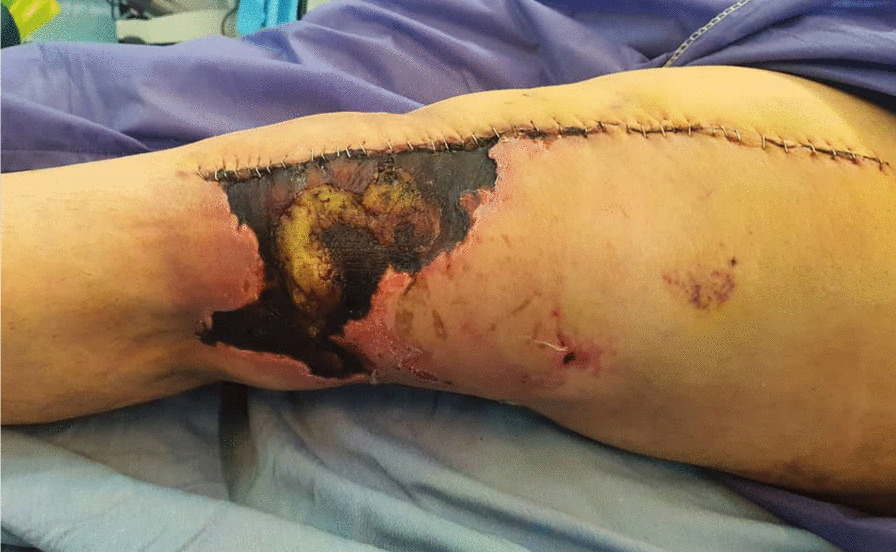
Skin necrosis in a patient of the control group

Skin necrosis occurred in one patient in the control group. There was no case of skin complication in the intervention group; however, the two groups had no significant difference in this respect (P-value = 0.31). This patient was a 60-year-old man who underwent surgery for a fracture the first time and TKA for the second time. The patient had no history of underlying disease. The length of the scar was 11.06 cm. The mentioned patient had a burn scar. (Fig. 3). Other wound complications were not observed in any patients. Also, no cases of infection were reported in either group.

### Surgery outcome

Six months after the operation, the mean KSS of the patients was 83.2 ± 10.2 in the intervention group and 82.9 ± 11.1 in the control group. This difference was not statistically significant (P-value = 0.33). There was no significant difference between the variables of bleeding rate, hemoglobin level, and duration of surgery in the two groups (Table 2).

**Table 2:** Surgery outcome in the two groups

Variable	< 8 cm distance between incisions (n = 25)	> 8 cm distance between incisions (n = 25)	P-value
Knee Society Score (after surgery)	82.9 (11.1)	83.2 (10.2)	0.29
Surgery time (minutes)	87.4 (20.33)	85.6 (19.63)	0.75
Bleeding during surgery (cc)	56.33 (16.88)	59.11 (18.36)	0.11
Hemoglobin (g/dl)	11.33 (4.30)	10.56 (3.66)	0.59

## Discussion

This study evaluated how making a new incision regardless of the location of a single previous incision scar affects developing wound complications, e.g., skin necrosis. According to our results, the rate of skin necrosis was not significantly different between the patients with a new incision made regardless of the location of the previous incision and those with a new incision 8 cm distant from the previous one. The rate of other wound complications also did not significantly differ. The functional outcome of the two groups was comparable, as well.

Skin necrosis is one of the devastating complications following TKA, rapidly predisposing the periprosthetic components to infection [15]. Therefore, reducing the risk of post-TKA skin necrosis is of vital essence. Previous incision scars are considered a risk factor for skin necrosis after TKA [16]. Thus, keeping a distance of almost 8 cm from the previous scar is suggested [9]. However, this distance may make it difficult to access the desired structures in the knee.

Ries evaluated the characteristics of skin necrosis in nine patients following TKA, and eight patients had a predisposing factor for developing skin necrosis, including prior skin scar, grafting, or contusion in four patients. They concluded that factors affecting local vascularity to the soft tissues, e.g., previous scars, peripheral vascular disease, steroid use, immunosuppressive disorders, and malnutrition, increase the risk of skin necrosis following TKA [16]. In the study of Shen et al., two out of seven patients who developed skin necrosis after TKA had a previous scar near the knee joint [17]. In the present study, skin necrosis only occurred in one patient, in whom the new incision had 8 cm distant from the previous scar. This inconsistency suggests the role of other confounding factors in the association of previous scar and skin necrosis, such as correct wound-edge alignment and tension-free closure [9].

Characteristics of the previous scar also affect the development of skin necrosis following the TKA. While utilizing the previous single scar is suggested to reduce the necrosis rate following the TKA [18], more lateral incisions are recommended when there are multiple previous scars. Thereby, leaving skin perfusion that originates medially intact reduces the rate of post-TKA skin necrosis [19]. A well-healed transverse incision does not seem to cause any complication; therefore, it can be ignored and crossed at right angles [20]. Using the previous scar for making a new incision was not possible in any of the patients. Patients with multiple scars were excluded as they had a higher risk of skin necrosis following the TKA. Patients with transverse scars were also excluded since transverse scars are not generally considered a risk factor for skin necrosis after TKA [20].

In a retrospective study, Zyl et al. evaluated if the presence of a previous scar increases the risk of skin necrosis in TKA. In total, six cases with minor wound edge slough occurred in their 925 (0.6%) patients. Three of these patients were in the group with a previous scar (n = 442), and the other three had no previous scar (n = 483). They concluded that the new scar should include the previous scar if they are both approximately in the line of a normal midline TKA incision. Otherwise, the previous scar can be ignored without any concern regarding the increased risk of skin necrosis [4]. None of the previous scars were in the line of a normal midline TKA incision in the present study. Therefore, a new incision was made by ignoring the location of a previous scar. Consistent with the results of Zyl et al., this strategy did not increase the risk of skin necrosis following the TKA.

Altogether, our results suggest that in patients undergoing TKA, the previous scar does not increase the risk of skin necrosis, and the traditional idea of observing the distance of 8 cm from the previous scar might be questioned.

This study was not without limitations. As the main limitation, the follow-up period of patients in the study was short. Also, our exclusion criteria were selective, which can affect the external validity of the study. Moreover, our sample size was sufficient for carrying out the study, however, a higher sample size can add to the power of the study. Therefore future studies with longer follow-up periods and higher sample sizes are suggested to confirm the results of the present study.

## Conclusions

The rate of skin necrosis may not significantly differ between the otherwise healthy TKA patients whose new incision is made regardless of the location of the previous incision scars and those in whom the new incision is made at an 8 cm distance from the previous scars. This observation suggests the possibility of ignoring the previous scar without concern regarding the increased risk of skin necrosis in patients undergoing TKA.

## Data Availability

The datasets generated and/or analyzed during the current study are not publicly available [part of a large study that has not yet been completed] but are available from the corresponding author on reasonable request.

## Abbreviations

TKA: Total knee arthroplasty
KSS: Knee Society Score
